# Acute bacterial meningitis in Iran: Systematic review and meta-analysis

**DOI:** 10.1371/journal.pone.0169617

**Published:** 2017-02-07

**Authors:** Hamidreza Houri, Ali Pormohammad, Seyed Mohammad Riahi, Mohammad Javad Nasiri, Fatemeh Fallah, Hossein Dabiri, Ramin Pouriran

**Affiliations:** 1 Department of Microbiology, School of Medicine, Shahid Beheshti University of Medical Sciences, Tehran, Iran; 2 Department of Epidemiology, Birjand University of Medical Sciences, Birjand, Iran; 3 Department of Epidemiology, Faculty of Health, Shahid Beheshti University of Medical Sciences, Tehran, Iran; 4 School of Medicine, Shahid Beheshti University of Medical Sciences, Tehran, Iran; Public Health England, UNITED KINGDOM

## Abstract

**Introduction:**

Bacterial meningitis persists in being a substantial cause of high mortality and severe neurological morbidity, despite the advances in antimicrobial therapy. Accurate data has not been available regarding the epidemiology of bacterial meningitis particularly in developing countries, yet. Indeed, the present systematic review provides a comprehensive data analysis on the prevalence and epidemiology of bacterial meningitis in Iran.

**Methods:**

We systematically reviewed articles from 1994 to 2015. The reports which contained the prevalence and etiology of acute bacterial meningitis by valid clinical and laboratory diagnosis were comprised in the present study.

**Results:**

Our analysis indicated that *Streptococcus pneumoniae* (30% [I2 = 56% p < 0.01]), *Haemophilus influenza* type b (15% [I2 = 82.75% p < 0.001]), coagulase negative staphylococci (CoNS) (14% [I2 = 60.5% p < 0.06]), and *Neisseria meningitidis* (13% [I2 = 74.16% p < 0.001]) were the most common cause of acute bacterial meningitis among meningitis cases in Iran. Notably, high frequency rates of nosocomial meningitis pathogens were detected in the present analysis.

**Conclusions:**

It was magnificently attained that the majority of cases for bacterial meningitis in Iran could be avertable by public immunization schemes and by preventive care to inhibit the broadening of hospital acquired pathogens.

## Introduction

Infectious meningitis includes viral, bacterial, fungal, and parasitic meningitis which have been considered as severe and potentially fatal pathogens, caused 422,900 deaths and according to the statistic in 2010 approximately 2628,000 patients with disabling sequelae [1]. In the meantime, acute bacterial meningitis (ABM) has been perceived as a common life threatening infection, especially in neonates and infants [2]. Even though, the introduction of broad-spectrum of antibiotics have made bacterial meningitis curable; nonetheless, the mortality and morbidity rate of this life threatening pathogens remains ostensibly high. Whereas, endemic bacterial meningitis residues relatively an infrequent illness in developed countries, the probability of occurrence of endemic and epidemic bacterial meningitis in undeveloped countries accumulates as the major infection [3]. As a matter of fact, ABM continues to be a principal reason of death among neonates in developing countries. Apparently, it seems that this disease debris’s the fourth leading cause of disability in these parts of the globe [4, 5]. Diagnosis of ABM is based on the combination of typical clinical symptoms and the consequent of laboratory tests which indicates the inflammatory response in cerebrospinal fluid (CSF); indeed, demonstrates the specific causative bacterial agent (Gram’s stain, culture, antigen assay and molecular detection) [6]. Three major pathogens have been detected for manifestation of ABM which are listed as follows: *Streptococcus pneumonia* (pneumococcus), *Haemophilus influenzae* type b (Hib), and *Neisseria meningitidis* (meningococcus). Transparently, they have been responsible for 118.400, 83.000, and 75.000 deaths, respectively worldwide [7, 8]. According to the above evidence analysis, a wide range of bacteria can cause ABM. Limpidly, group B Streptococci (GBS) or *Streptococcus agalactiae* is one of the primary causes of neonatal bacterial meningitis in premature and term infants up to 3 months in many developing countries [9]. The other transpicuous deliberated bacterial strains which apparently could cause bacterial meningitis are Enterobacteriaceae spp., principally *Escherichia coli* K1 and *Klebsiella pneumoniae* that can be quite fatal. Moreover, *Listeria monocytogenes* has been rarely responsible for bacterial meningitis in newborns and infants, especially during zoonotic outbreaks [10, 11].

Translucently, It is essential to state that according to the previous repots, the prevalence and etiologies of ABM vary in each geographical area [12]. To the best of our knowledge, there is no sufficient data regarding the epidemiology of bacterial meningitis in Iran. Allegedly due to the lack of a routine vaccination program against meningeal pathogens; such as, pneumococci in Iran, ABM is considered as a public health hazard with high rate of morbidity, mortality, and tremendous cost burden for the health care providers [13]. Consequently, since there was no accurate estimate of the burden of ABM in Iran, the present systematic study was conducted to provide useful insights concerning the prevalence and epidemiology of the ABM in Iran. Systematic review and meta-analysis according to the Preferred Reporting Items for Systematic reviews and Meta-Analyses (PRISMA) statement were used (S1 Table) [14].

## Materials and methods

### Search strategies

From January 1, 1994 to May 1, 2015, the entire studies addressing meningitis infections in Iran were collected from world-wide databases including Medline (via PubMed), Web of Science, Embase, and Iranian national databases. Presumably, the search was carried out in both English and Farsi, distinctly and the research was restricted solely to original articles. The following search terms containing Medical Subject Headings (MESH) or keywords in text, title, or abstract were used with the help of Boolean operators (“and” or “or”): ‘‘bacterial”, ‘‘meningitis” and ‘‘Iran”. In addition to English articles, all relevant papers in national databases; included Scientific Information Database (www.sid.ir) and Magiran (www.Magiran.com), were utilized with similar search strategy and Farsi keywords. The reference and citation lists for the regain papers were searched by this phenomenal strategy and with any selected additional study.

### Inclusion and exclusion criteria

Apparently, the entire original articles presenting cross-sectional studies on the prevalence and etiology of ABM in patients with suspected meningitis in Iran were considered. Diagnosis of meningitis in the included studies were performed by abnormal CSF findings, including: increased polymorphonuclear leukocytes counts, protein concentration more than 100 mg/dl and hypoglycorrhachia, and CSF culture as the gold standard method for detecting and identifying meningeal isolates [15]. Evidently, numerous studies were excluded from the analysis because of the following reasons: articles which only focused on individual etiological agent for meningitis; article which only considered sub-acute or chronic meningitis, those which only considered individual groups of patients such as HIV positive or immunocompromised patients, surveys which contained other infectious meningitis besides bacterial infections; such as, viral, fungal, and those papers which did not utilize the mentioned methods above; furthermore, reviews and systematic review articles, case reports, and articles which were only available in abstract form were excluded.

### Data extraction and definitions

Data were extracted using an extraction form independently and in duplicate by 2 investigators. The following data were extracted from the chosen articles: the first author’s name, the year of the study, the year of publication, the mean age, the location of the studies, the number of cases participated, the method of conducting surveys, the source of samples, sample size, and the prevalence of ABM. The studies identified by the search strategy and were reviewed for eligibility based on title and abstract by two authors. Differences in data extraction between investigators were resolved by consensus.

### Quality assessment

Perspicuously, the quality assessment of inquiries were accompanied by two reviewers independently according to the Critical Appraisal Checklist recommended by the Joanna Briggs Institute [16]; moreover, the disagreements were resolved by consensus. The checklist composed of ten questions in which the reviewers answered the questions for the chosen studies based on an individual basis. The ‘Yes’ answer for each question got a point; indeed, the scores ranged from zero to ten. Apparently, the studies which obtained more than 60% were included in the present inquiry.

### Meta-analysis

The statistical analysis was carried out by Stata (version13) software. The point estimates of the effect size, the prevalence of bacterial meningitis, and its 95% confidence interval (95% CI) were estimated peculiarly for each study. Random effects models were used to estimate pooled effect. In this regard, the heterogeneity among studies were tested by Cochran’s Q-statistic I2 squared and Galbraith graph. In order to estimate pooled effect, we utilized metaprop command rather than metan in Stata software. For installing, the command needs to be connected to the internet and then typing ssc install metaprop in Stata. We used ftt cimethod (score) or Freeman-Tukey transformation procedures for collecting binomial data. Contrary to metan command, Stata automatically omits studies which their proportion were 0 or 1, transparently, this method does not make the mentioned problem; eventually, plot confidence in forest intervals always were in the range of 0–1 [17–19]. The P-value, 0.05 was considered significant.

## Results

### Characteristics of included studies

**Fig 1:**
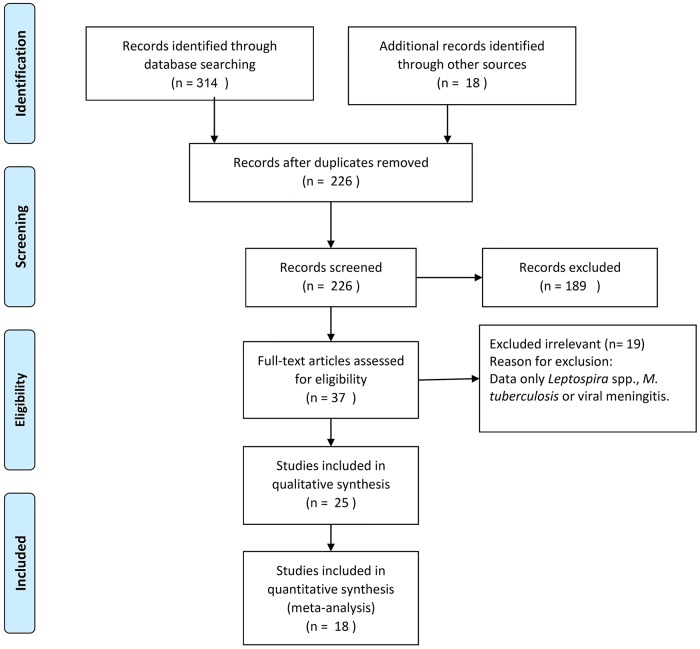
Flow diagram of literature search and study selection.

Translucently, a total of 314 reports were screened for the analysis of patients with ABM. Out of these studies 18 met the inclusion pellucid criteria (Fig 1). Characteristics of the selected articles are summarized in Table 1. In fact, 16 studies that were included in the analysis were in English and the rest were in Farsi. Overall, 1078 culture positive samples were analyzed in the included inquiries. Geographic location of the reports comprised east to west and north to south part of Iran; indeed, the majority of patients were coming from central part of Iran. The main diagnostic method in all included studies was the isolation and culture of the pathogen from CSF, or from blood and CSF. Moreover, in most of these studies, the plumb diagnosis was confirmed by additional techniques including serological and molecular assays.

**Table 1:** Characteristics of studies included in the meta-analysis.

Study	Published time	Enrollment time	Sampling area	Mean age	No. of culture positive
**Attarpour *et al*.** [30]	2014	2012–2013	Tehran	61±40.2 months	114
**Ataee *et al*.** [31]	2011	2005–2009	Tehran	16–70 years	171
**Qurbanalizadegan *et al*.** [32]	2010	2002–2006	Tehran	<5 years	36
**Motamedifar *et al*.** [33]	2015	2011–2013	Shiraz	34±26 years	225
**Yousefi *et al*.** [34]	2006	1998–2002	Hamedan	<5 years	146
**Abdinia *et al*.** [35]	2014	2003–2013	Tabriz	4.2 years	107
**Zamani *et al*.** [36]	2005	1994–1999	Tehran	10–28 days	15
**Ghotaslou *et al*.** [37]	2012	2008–2009	Tabriz	35±2 months	11
**Heydarian *et al*.** [38]	2014	2005–2012	Mashhad	12.7 months	5
**Ehsanipour *et al*.** [39]	2004	1997–2002	Tehran	28.5±18.3 months	4
**Haghiashteiani *et al*.** [40]	2008	2001–2007	Tehran	*	121
**Khalessi *et al*.** [41]	2014	2008–2012	Tehran	8.41 days	4
**Bagheri *et al*.** [42]	2015	2006–2012	Mazandaran	34.35±18.28 years	14
**Alavi *et al*.** [43]	2010	2003–2007	Ahvaz	44.7 ± 26.7 years	42
**Bahador *et al*.** [44]	2009	2003–2005	Kerman	15.2±5.2 years	12
**Rezaei *et al*.** [45]	2013	2008–2009	Tehran	45.34±42.2 years	11
**Aletayeb *et al*.** [46]	2010	1997–2007	Ahwaz	29 days	31
**Mahmoudi *et al*.** [47]	2013	2009–2011	Tehran	10 years	20

*Specimens collected from children hospital

### The prevalence of bacteria

As shown in the Table 2, pneumococcus (30% [I2 = 56% p < 0.01]), Hib (15% [I2 = 82.75% p < 0.001]), CoNS (14% [I2 = 60.5% p < 0.06]), and meningococcus (13% [I2 = 74.16% p < 0.001]) were the most common cause of culture positive ABM.

**Table 2:** Meta-analysis of proportion of different bacteria in culture positive cases.

Organism	Frequency of bacteria (%)	Confidence interval 95%	n/N	Heterogeneity Test, I2 (%)	Heterogeneity test, P value
*S*. *pneumonia* < 10 years old	36	31–40	67/311	0.00	0.94
*S*. *pneumonia* ≥10 years old	20	13–27	182/507	32.23	0.19
*S*. *pneumonia* Overall	30	24–30	249/818	56	0.01
*H*. *influenzae*	15	8–22	118/876	82.75	<0.001
CoNS	14	8–22	54/384	60.5	0.06
*N*. *meningitides*	13	7–20	70/673	74.16	<0.001
*Klebsiella spp*.< 1 month	4	2–7	15/50	0.00	0.94
*Klebsiella spp*.≥ 1 month	28	11–48	17/316	36.72	0.21
*Klebsiella spp*. Overall	9	3–18	32/376	71.53	<0.001
*E*. *coli*	8	6–11	57/936	20.75	0.26
Others	4	2–7	35/694	31.67	0.19

#### *Streptococcus* *pneumonia*

**Fig 2:**
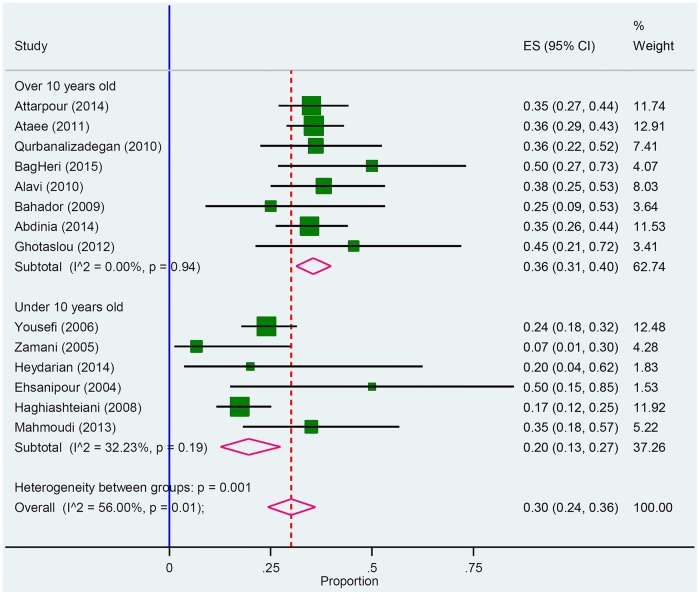
Forest plot of the meta-analysis on proportion of *S*. *pneumonia* in culture positive cases.

Distinctly, out of the 18 included studies; 14 studies met the inclusion criteria for Pneumococcus. As it was shown in Table 2 and Fig 2, Pneumococcus was responsible for 30% of ABM in Iran. Precisely, the review of the collected reports also manifested pneumococci was the most common causative agent for meningitis in 11 statistical analysis reports (61.11% of studies).

#### *Haemophilus* *influenza*

**Fig 3:**
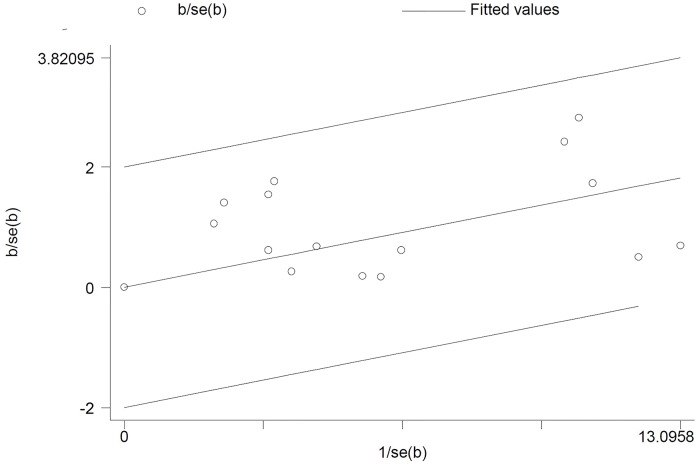
Galbrath plot of the meta-analysis on *H*. *influenza* in culture positive cases.

**Fig 4:**
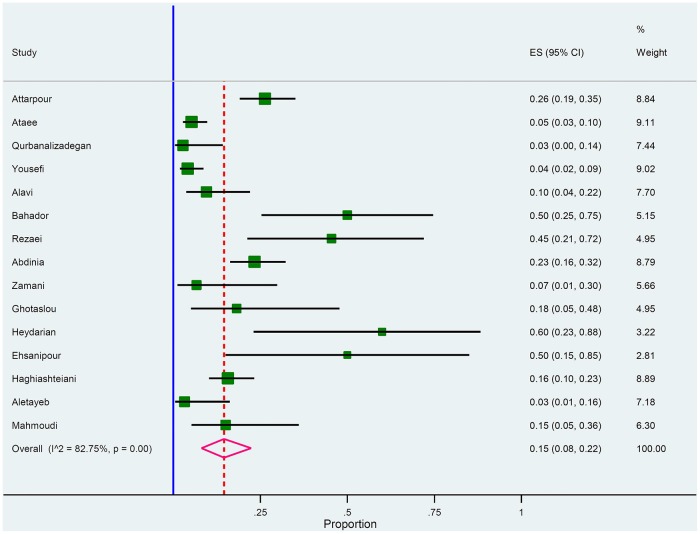
Forest plot of the meta-analysis on proportion of *H*. *influenza* in culture positive cases.

Articulately, out of the 18 included studies; 15 studies met the inclusion criteria for Hib (Fig 3). As it is presented in Galbraith plot in the Fig 4, the meta-analysis on *H*. *influenza* in culture positive meningitides; this graph shows heterogeneity among studies; in addition, all studies were in -2 to +2 z-score rang without any outlier point.

#### *Neisseria* *meningitides*

**Fig 5:**
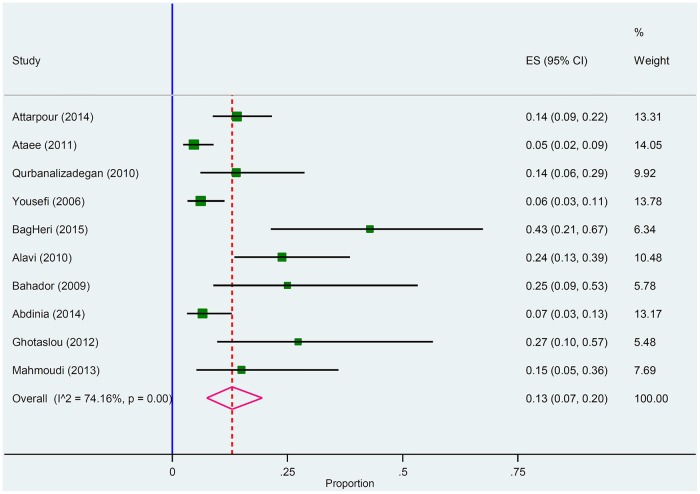
Forest plot of the meta-analysis on proportion of *N*. *meningitides* in culture positive cases.

**Fig 6:**
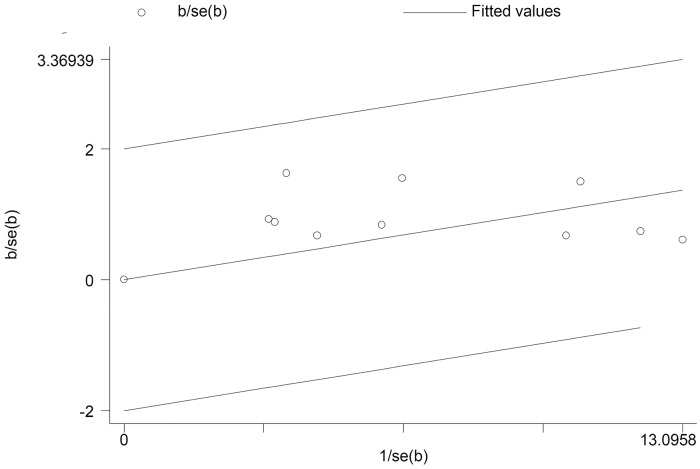
Galbrath plot of the meta-analysis on *N*. *meningitides* in culture positive cases.

Of these 18 included studies; 10 studies met the inclusion criteria for *N*. *meningitides* (Fig 5). Galbrath plot of the meta-analysis on *N*. *meningitides* in culture positive meningitides showes heterogeneity among reports and all studies were in the range of -2 to +2 z-score without any outlier point (Fig 6).

### Clinical and laboratory features

Overall, 43% (95% CI 30–57%) of neonate with CSF-culture positive were preterm (Table 3). Furthermore, 39% (95% CI 26–52) of the utter patients suffered from malnutrition. The clinical presentation of ABM was characterized by fever (71%, CI 37–69%), irritability (60%, 95% CI 52–69%), and seizures (33%, 95% CI 25–41%). In addition, more clinical features are shown in Table 4. It is mentionable that laboratory features in CSF-culture positive samples were characterized by increased CSF leukocytes counts (753.353 (cells/mm3), 95%CI 596.294–910.412), increased CSF protein (415.5 (mg/dl), 95% CI 86.5–744), decreased CSF glucose (30.46 (mg/dl), and 95% CI 25.17–35.74) (Table 3).

**Table 3:** Meta-analysis of proportion of clinical and laboratory features in culture positive meningitis.

**Clinical features**	**Proportion in culture positive meningitis (%)**	**Confidence interval 95%**	**n/N**	**Heterogeneity test, I2 (%)**	**Heterogeneity test, P value**
Preterm (in neonate)	43	30–57	29/66	18.77	0.29
Malnutrition	39	26–52	63/181	43.79	0.17
Seizures	33	25–41	104/320	35	0.17
Irritability	60	52–69	77/127	0.00	<0.001
Fever	71	37–96	261/312	96.11	<0.001
**Laboratory features**	**Prevalence in culture positive meningitis**		**n/N**	**Heterogeneity Test, I2 (%)**	**Heterogeneity test, P value**
Protein (mg/dl)	415.5	86.5–744	-	99.4	<0.001
Glucose (mg/dl)	30.46	25.17–35.74	-	36.58	<0.001
CSF leukocyte count (cells/mm3)	753.353	596.294–910.412	-	0.07	0.793

**Table 4:** Clinical characteristics of patients with bacterial meningitis.

Clinical presentation	
Decreased Moro Reflex	20/5 (25%)
Icter	2/20 (10%)
Hydrocephaly	61/ 186 (32.8%)
Brain edema	5/40 (12.5%)
Subdural Effusion	5/40 (12.5%)
Lethargy	51/157 (32.5%)
Nausea and vomiting	179/268 (66.8%)
Headache	85/ 253 (33.6%)
Leukocytosis	109/146 (74.65%)
Died	23/197 (11.6%)

## Discussion

Inclusively, the survey which was obtained from this investigation indicated that pneumococcus was the most common etiologic agent determined in patients with ABM in Iran and likewise, Hib, CoNS, and meningococcus were diagnosed respectively. Causative organisms for bacterial meningitis differ according to the population investigated, age of the participants, and geographic area in which the data were analyzed [20]. We considered the entire mentioned variables correlations with the prevalence of ABM causative agents in our analysis; nonetheless, the statistical significant variables like age solely were reported.

In the recent decade, pneumococcus has been considered as the main causative agent for bacterial meningitis in children less than 10 years old, in elder population, and in immunocompromised hosts in the United States as well as in European countries [21]. Indeed, the present study conducted a meta-analysis for the prevalence of pneumococcus meningitis in Iran. According to the statistical analysis, the prevalence of pneumococcus meningitis in children less than 10 years and individuals over 10 years old was 36% (95% CI; 31–40) and 20% (95% CI; 13–27) thereof. Peculiarly, there was a significant difference in the prevalence of these two sub-groups. The high prevalence rate of pneumococcal meningitis in Iran is alarming since the emergence of drug resistant strains; especially those are resistant to penicillin and third-generation cephalosporins. Penicillin resistance is an indicator of reduced susceptibility to other antibiotic agents, which could lead to treatment failures in patients with pneumococcal meningitis [22]. Moreover, the 7-valent pneumococcal conjugate vaccine (PCV-7) became commercially available; however, it is not as a mandatory prescribed vaccine in Iran and is only prescribed for high-risk individuals. Therefore, pneumococcal meningitis could have the potential to become a serious life threatening infection in this region.

According to the analysis retrieved, Hib (15%) was the second most common causative agent for bacterial meningitis in Iran. It is quite essential to mention that before the invention of the conjugate polysaccharide vaccine against Hib, this pathogen was the most incitement of meningitis world-wide. It is mentionable that as of now Hib meningitis is peculiarly rare in areas with routine Hib vaccination [23]. In Iran, based on the CDC reports, the rate of Hib meningitis was <15 per 100,000 in 2004 [24]. Moradi *et al*. predicted that vaccination against Hib in 2008 in the birth cohort in Iran could reduce from 385 meningitis cases to 62 cases [25]. Accordingly, there are facts that after 2014, the major achievement of the immunization programs occurred in Iran following the introduction of the DTPw-HB/Hib prevalent vaccine (containing diphtheria-tetanus-whole cell pertussis-hepatitis B/Hib); moreover, a significant reduction in the mortality and morbidity rate of Hib meningitis is predictable due to Hib meningitis in Iran. It should be noted that all the included studies in the present analysis were conducted before 2014.

Notably, CoNS was the third most common cause of ABM according to our analysis which accounted for 14% (95% CI; 8–22) of the entire cases. Nosocomial bacterial meningitis poses a considerable public health hazard because of the growing emergence of multidrug resistant organisms (MDROs) and severely limited treatment options [26, 27]. As a matter of fact, the high incidence of nosocomial meningitis reported in Iran is alarming. It is essential to pay attention for controlling and preventing CNS infections by virtue of the opportunistic organisms. Now it's time to make the decision to improve the quality of infection control systems and practices in Iranian health centers according to international guidelines.

Meningococcus was considered as another common cause of ABM in Iran which accounts for 13% (95% CI; 7–20) of all cases. The only reported document regarding the meningococcal incidence rate in Iran was conformed in 2005, the search was done from 1996 until now, when the rate was 0.14 per 100 000 [28]. In 2000, meningococcal caused by the serogroup W-135 outbreak strain associated with the Hajj pilgrimage were reported in certain Middle Eastern and North Africa countries, including Iran [29]. Indeed, explicitly there is no sufficient data concerning the incidence rate of meningococcal meningitis in Iran. A polyvalent meningococcal vaccine, containing the purified polysaccharide capsules of group A, C, Y, and W-135 meningococci, is accessible in Iran. Nevertheless, the vaccine is not mandatory for the population, solely it is required for those who travel abroad (especially to Saudi Arabia for Hajj) and enlisted soldiers. Therefore, public immunization against meningococcus can reduce prevalence of ABM due to this pathogen.

Clinical features give the clue to the early diagnosis of ABM by a physician; therefore, there are various clinical symptoms which can be quite helpful in the diagnosis process. In the recent study, 71% (95% CI; 37–96) of ABM patient manifested sign and symptoms of fever with high heterogeneity (I2 = 96.11% p < 0.001). by using medications which are not prescribed by medical doctors for fever or other symptoms can head to heterogeneity and drug resistance consequences. The analysis showed that preterm 43% (95% CI; 30–57) had a distinct risk factor for neonate ABM. The other predominant finding in this study was showing the early screening techniques and the analysis of CSF for early screening of ABM (Tables 3 and 4). There was no significant relationship between alcoholism, splenectomy, immunosuppressive medication, and diabetes mellitus with culture positive patients. In future studies more inquiries are needed for other aspect of bacterial meningitis like the pattern of drug resistance, prevention, rapid, and novel detection methods because of the emergency of bacterial meningitis.

## Conclusions

The current systematic review indicated that the most common causative agent of ABM could be vaccine-preventable pathogens in Iran. Further accurate and efficient data regarding the prevalence of the etiological agents for bacterial meningitis, especially after public immunization against Hib, can be exploited for an effective immunization schedules. Indeed, to reduce the incidence of nosocomial meningitis, prevention and control measures should be considered in accordance to the international standards in Iranian clinics and hospitals.

## Supporting information

S1 Table PRISMA checklist. (DOC)
